# Identification and Characterization of a Novel *Issatchenkia orientalis* GPI-Anchored Protein, IoGas1, Required for Resistance to Low pH and Salt Stress

**DOI:** 10.1371/journal.pone.0161888

**Published:** 2016-09-02

**Authors:** Akinori Matsushika, Kanako Negi, Toshihiro Suzuki, Tetsuya Goshima, Tamotsu Hoshino

**Affiliations:** 1 Research Institute for Sustainable Chemistry (ISC), National Institute of Advanced Industrial Science and Technology (AIST), Hiroshima, Japan; 2 Graduate School of Advanced Sciences of Matter, Hiroshima University, Hiroshima, Japan; 3 National Research Institute of Brewing (NRIB), Hiroshima, Japan; National Renewable Energy Laboratory, UNITED STATES

## Abstract

The use of yeasts tolerant to acid (low pH) and salt stress is of industrial importance for several bioproduction processes. To identify new candidate genes having potential roles in low-pH tolerance, we screened an expression genomic DNA library of a multiple-stress-tolerant yeast, *Issatchenkia orientalis* (*Pichia kudriavzevii*), for clones that allowed *Saccharomyces cerevisiae* cells to grow under highly acidic conditions (pH 2.0). A genomic DNA clone containing two putative open reading frames was obtained, of which the putative protein-coding gene comprising 1629 bp was retransformed into the host. This transformant grew significantly at pH 2.0, and at pH 2.5 in the presence of 7.5% Na_2_SO_4_. The predicted amino acid sequence of this new gene, named *I*. *orientalis GAS1* (*IoGAS1*), was 60% identical to the *S*. *cerevisiae* Gas1 protein, a glycosylphosphatidylinositol-anchored protein essential for maintaining cell wall integrity, and 58–59% identical to *Candida albicans* Phr1 and Phr2, pH-responsive proteins implicated in cell wall assembly and virulence. Northern hybridization analyses indicated that, as for the *C*. *albicans* homologs, *IoGAS1* expression was pH-dependent, with expression increasing with decreasing pH (from 4.0 to 2.0) of the medium. These results suggest that *IoGAS1* represents a novel pH-regulated system required for the adaptation of *I*. *orientalis* to environments of diverse pH. Heterologous expression of *IoGAS1* complemented the growth and morphological defects of a *S*. *cerevisiae gas1Δ* mutant, demonstrating that *IoGAS1* and the corresponding *S*. *cerevisiae* gene play similar roles in cell wall biosynthesis. Site-directed mutagenesis experiments revealed that two conserved glutamate residues (E161 and E262) in the IoGas1 protein play a crucial role in yeast morphogenesis and tolerance to low pH and salt stress. Furthermore, overexpression of *IoGAS1* in *S*. *cerevisiae* remarkably improved the ethanol fermentation ability at pH 2.5, and at pH 2.0 in the presence of salt (5% Na_2_SO_4_), compared to that of a reference strain. Our results strongly suggest that constitutive expression of the *IoGAS1* gene in *S*. *cerevisiae* could be advantageous for several fermentation processes under these stress conditions.

## Introduction

To date, the global consumption of energy has been primarily dependent on finite non-renewable fossil fuels (petroleum). It is believed that the continued use of fossil fuels at the current rate will lead to an increase in global warming and cause more severe climate change; biofuels and bioproducts produced from biomass are increasingly viewed as a viable approach to prevent this scenario [1,2]. Bioethanol, an eco-friendly renewable liquid fuel, is currently the most widely used biofuel [3] but is generated from sugar-containing and starchy biomass, such as corn grain and sugarcane, which are edible agricultural products. Consequently, bioethanol production competes with the availability of food supplies, causing serious problems [4]. Non-edible lignocellulosic biomass sources such as wood and agricultural residues are therefore the expected sources of sugar in second-generation bioethanol production processes. Lignocellulosic biomass is pretreated and hydrolyzed to obtain potentially fermentable monomeric sugars, mainly glucose and xylose [5]. The budding yeast *Saccharomyces cerevisiae*, which efficiently produces ethanol from hexose sugars, including sucrose and glucose, is the most commonly used microorganism in industrial ethanol production. Because *S*. *cerevisiae* cannot ferment the pentose sugar xylose, there has been extensive effort to develop genetically engineered strains capable of fermenting xylose and glucose simultaneously [6]. However, biomass pretreatment produces hydrolysates that are saline, have high osmotic pressure [7], and contain high concentrations of inhibitors [8], all of which negatively influence fermentation by yeast. In addition, yeast cells are exposed to various other environmental stresses during ethanol production, including acid stress [9,10], high concentrations of ethanol [11,12], and high temperature [13,14]. Therefore, the development of robust strains with high stress tolerance is crucial for reducing cooling and ethanol recovery costs, and for minimizing the risk of contamination during commercial ethanol production [15–17].

Acid stress is a critical problem in several bioproduction processes, including lignocellulosic ethanol production by yeast. Low pH inhibits cell proliferation and viability as a result of the increased proton gradient across the plasma membrane [18]. Although the best pretreatment methods and fermentation conditions depend greatly on the type of lignocellulosic biomass, acid-based pretreatment methods using dilute acid or SO_2_ are widely used for the production of ethanol at the industrial level [19]. This pretreatment results in residual acid, and therefore a neutralization step is required prior to downstream enzymatic hydrolysis or fermentation. However, if the yeast strains used in the fermentation process could produce a significant amount of ethanol under acidic conditions, the neutralization step could be simplified or omitted, leading to decreased neutralization costs. In addition, ethanol fermentation by acid-tolerant yeast under acidic conditions below pH 4.0 minimizes the risk of bacterial contamination and reduces the cost of sterilization [20,21]. Tao et al. reported no contamination during ethanol fermentation by an acid-tolerant mutant of *Zymomonas mobilis* under non-sterile conditions below pH 4.5 [22]. Moreover, the addition of lactate to the culture medium of the lactate-tolerant yeast *Candida glabrata* inhibited the growth of contaminant *Lactobacillus* strains without affecting ethanol production by the yeast strain [23]. Enhanced tolerance to acid stress is an indispensable advantage for lactic acid production using lactic acid bacteria because the pH of the culture medium decreases due to the accumulation of lactic acid, thereby affecting cell growth and hence bacterial production of lactic acid. Although *S*. *cerevisiae* is more resistant to low pH than are lactic acid bacteria, the yield of lactic acid nonetheless decreases when fermentation is conducted below pH 2.8 using a recombinant *S*. *cerevisiae* strain [24]. Accordingly, conferring higher acid tolerance to *S*. *cerevisiae* would improve both ethanol and lactic acid production under acidic conditions [25].

Following acid-based pretreatment processes, the residual acid is neutralized with alkalis such as NaOH, thereby producing salts such as Na_2_SO_4_ [26]. Salts also can originate from the biomass [8]. A number of salts, which are ionic compounds composed of cations and anions, have significant inhibitory effects on fermentation. Notably, salts are known to reduce the cell growth, sugar consumption rates, and ethanol production rates of *S*. *cerevisiae*, thereby increasing the amount of by-products such as glycerol [27–29]. Fermentation by salt-tolerant yeasts under high salt concentrations would therefore reduce the cost of desalting and decrease the possibility of contamination. Recently, Casey et al. showed that salts affect xylose utilization more strongly than glucose utilization [30]. These results can be explained by two inhibition processes: intracellular ion toxicity and osmotic stress [31,32]. Hence, the use of yeasts tolerant of both salt and acid stress is of industrial importance for bioproduction processes using lignocellulosic biomass.

The acid- and salt-tolerant yeast *Issatchenkia orientalis* (syn. *Pichia kudriavzevii*) has been isolated from a variety of environments, including sourdough [33], cocoa bean [34], cornstalk, sweet sorghum stalk, and rice straw [35]. The *I*. *orientalis* MF-121 strain was isolated from a highly acidic river (pH 3.0) flowing in a hot spring area in Japan and can ferment glucose to ethanol in acidic media (pH 2.0) containing 5% Na_2_SO_4_ [36,37]. In contrast, *S*. *cerevisiae* strains grow and ferment poorly in these acid-salt media [36,37]. Furthermore, the MF-121 strain can produce ethanol using saccharification products hydrolyzed from lignocellulosic biomass using sulfuric acid [38]. It has also been reported that *I*. *orientalis* is tolerant to several other fermentation inhibitory compounds such as furan derivatives (e.g., furfural [35] and 5-hydroxymethylfurfural (5-HMF) [39]) and organic acids (e.g., acetic acid [34,40] and formic acid [34]). The factors that affect the growth and fermentation of this yeast under low-pH conditions may play an important role in protecting against the lethal effects of weak acids. *I*. *orientalis* is clearly a multiple-stress-tolerant yeast but can only ferment glucose, fructose, and mannose to ethanol [36,40]. Furthermore, the tools and technologies for genome engineering of *I*. *orientalis* are extremely limited, perhaps due to the extra technical difficulties in the genetic manipulation of its genome [41]. Nevertheless, Kitagawa et al. constructed a transformation system for *I*. *orientalis* and succeeded in expressing a heterologous gene [42]. However, the genes related to stress tolerance (e.g., low pH and salt) in *I*. *orientalis* have not yet been reported, despite its importance for bioprocess applications.

To gain new insight into the mechanisms underlying low-pH- and salt-stress tolerance, and to improve the stress tolerance of *S*. *cerevisiae* cells, the present study screened for *I*. *orientalis* genes that can confer a low-pH- and salt-tolerant phenotype in *S*. *cerevisiae*. We isolated and identified a novel gene by screening a genomic library of *I*. *orientalis* constructed in this study. This gene, which we have designated *IoGAS1*, appears to be highly homologous to the *GAS* family of *S*. *cerevisiae*, whose members encode glycosylphosphatidylinositol (GPI) -anchored glycoproteins [43]. *S*. *cerevisiae GAS1* encodes a key enzyme in yeast cell wall assembly and to date is the best characterized member of the *GAS* family [44,45]. We found that overexpression of *IoGAS1* in *S*. *cerevisiae* significantly improved growth and fermentation at low pH values and high salt concentrations. We further characterized a potential role of the *IoGAS1* gene in fungal cell wall integrity, as assessed by the ability of *IoGAS1* to complement the growth and morphological phenotype of a *S*. *cerevisiae gas1* knockout strain. We also compared the gene expression profiles of an *I*. *orientalis* strain grown under different pH conditions and found that *IoGAS1* displays a unique expression pattern. Our results are discussed with respect to the presumed function of *IoGAS1*.

## Materials and Methods

### Yeast strains and media

The *Escherichia coli* strains HST08 (Takara Bio, Kyoto, Japan) and DH5α were used for cloning and were grown in Luria-Bertani (LB) broth or on agar plates (10 g/L tryptone, 5 g/L yeast extract, 5 g/L sodium chloride). The *E*. *coli* transformants were cultivated in LB medium supplemented with ampicillin (100 mg/L). The strain *I*. *orientalis* NBRC1279, a wild-type strain from the NITE Biological Resource Center in Chiba, Japan, provided the genomic DNA used to construct the genomic library. This strain was also used for gene expression studies using northern blotting. The *S*. *cerevisiae* strains used in this study are listed in Table 1. The wild-type *S*. *cerevisiae* strain BY4742 was adopted as a host for transformation with the *I*. *orientalis* genomic library and was used for cloning the library plasmids. The *gas1Δ* knockout strain in a BY4742 strain background (Open Biosystems, Huntsville, AL, USA) was used as the recipient yeast strain for overexpression of the *IoGAS1* gene and mutated *IoGAS1* genes.

**Table 1:** *S*. *cerevisiae* strains and plasmids used in this study.

Strain / plasmid	Relevant genotype	Source / reference
*S*. *cerevisiae* strains		
BY4742	*MATα his3Δ1 leu2Δ0 lys2Δ0 ura3Δ0*	Open Biosystems
B4-CON	BY4742, pPGK	This study
B4-CL1	BY4742, pPGK-CL1	This study
B4-IoPXR1	BY4742, pPGK-IoPXR1	This study
B4-IoGAS1	BY4742, pPGK-IoGAS1	This study
*gas1Δ*	Yeast knockout strain (YKO_GAS1)	Open Biosystems
D1-CON	*gas1Δ*, pPGK	This study
D1-IoGAS1	*gas1Δ*, pPGK-IoGAS1	This study
D1-IoGAS1(E161Q)	*gas1Δ*, pPGK-IoGAS1(E161Q)	This study
D1-IoGAS1(E262Q)	*gas1Δ*, pPGK-IoGAS1(E262Q)	This study
Plasmids		
pPGK	*URA3*	[46]
pPGK-CL1	*URA3*; expression of *IoPXR1* and *IoGAS1* genes	This study
pPGK-IoPXR1	*URA3*; expression of *IoPXR1* gene	This study
pPGK-IoGAS1	*URA3*; expression of *IoGAS1* gene	This study
pPGK-IoGAS1(E161Q)	*URA3*; expression of *IoGAS1* gene with E161Q mutation	This study
pPGK-IoGAS1(E262Q)	*URA3*; expression of *IoGAS1* gene with E262Q mutation	This study

The wild-type *I*. *orientalis* and *S*. *cerevisiae* strains were grown in yeast peptone dextrose (YPD) broth or on agar plates (10 g/L yeast extract, 20 g/L peptone, 20 g/L glucose) unless otherwise noted. For northern blot analysis, *I*. *orientalis* NBRC1279 was grown anaerobically in YPD medium adjusted to different acidic pH values (4.0, 3.0, 2.5, and 2.0). Recombinant *S*. *cerevisiae* strains (B4-CON, B4-CL1, B4-IoPXR1, B4-IoGAS1, D1-CON, D1-IoGAS1, D1-IoGAS1(E161Q), and D1-IoGAS1(E262Q)) (see Table 1) were grown in synthetic complete (SC) minimal medium (6.7 g/L yeast nitrogen base without amino acids) supplemented with the appropriate amino acids and nucleic acids [47] and 20 g/L glucose (SCD medium). In anaerobic fermentation experiments using recombinant *S*. *cerevisiae* strains, glucose (45 g/L) was added to the SC medium (SCD-2 medium). The pH values of the SC-based media (SCD and SCD-2) were adjusted to 2.5, 2.2, and 2.0 by the addition of H_2_SO_4_. For experiments involving sodium sulfate, appropriate amounts (5% or 7.5%) of sodium sulfate were added to the SC-based medium prior to pH adjustment; the pH of the medium was then adjusted with H_2_SO_4_. Unless specified, the yeast strains were grown at 30°C, either on agar plates or in liquid medium.

### Construction and amplification of the genomic library

*I*. *orientalis* (NBRC1279) genomic DNA used for construction of the genomic library was prepared as described previously [48]. The DNA was randomly sheared mechanically and fractionated by agarose gel electrophoresis. DNA fragments greater than 3–5 kb were purified from the agarose gel and treated using a Mighty Cloning Reagent Set (Blunt End) (Takara Bio) to convert both ends into blunt ends. The blunt-ended DNA fragments were ligated with bacterial alkaline phosphatase (BAP) -treated pPGK shuttle vector [46] (a multicopy (2-μm DNA-based) plasmid containing the *URA3* gene as a selective marker for yeast cells) that had been digested by *Hind*III followed by T4 DNA polymerase treatment. For amplification of the genomic libraries, the ligation mixture was introduced into *E*. *coli* HST08 competent cells (Takara Bio) by electroporation at 25 μF and 1.8 kV with the pulse controller set to 200 Ω on a GenePulser (Bio-Rad Laboratories, Hercules, CA, USA). Cells from ampicillin-resistant colonies were recovered from the plates and pools of plasmid DNA were prepared. The titers of the amplified libraries were approximately 1.5 x 10^8^ colony-forming units (cfu) per milliliter (cfu/mL) of the amplified library.

### Yeast functional screening and isolation of an acid-resistant clone

The genomic library from *I*. *orientalis* (NBRC1279) was transformed into *S*. *cerevisiae* (BY4742) using the lithium acetate method [49]. A total of 8.6 × 10^4^ clones of *S*. *cerevisiae* transformants expressing *I*. *orientalis* proteins were isolated and plated onto SCD agar, pH 2.0, to select for acid-tolerant yeast transformants. The screening plates were incubated at 30°C. The host strain did not grow under these conditions of acid stress, whereas the NBRC1279 strain did grow. After 20 days’ incubation, the single colony that grew was picked and streaked on a fresh SCD plate for further analysis. This putative positive yeast clone was denoted B4-CL1. Plasmid DNA from the B4-CL1 strain was isolated using a Speedprep yeast plasmid isolation kit (Dualsystems Biotech, Schlieren, Switzerland) and sequenced from both ends of its inserted DNA fragment according to the manufacturer’s recommended protocol (BigDye Terminator v3.1 Cycle Sequencing kit; Applied Biosystems, Foster City, CA, USA). Using the GENETYX (ver. 9.1) gene analysis software (Genetyx Corp., Tokyo, Japan), the nucleotide sequence of the clone insert was analyzed for the presence of open reading frames (ORFs), which then were translated to yield predicted amino acid sequences. The sequences of the predicted proteins were assessed (using the BLASTP algorithm) for homology to GenBank fungal protein sequences in the non-redundant sequence database (National Center for Biotechnology Information [NCBI]) to analogize the functions of the cloned genes.

### Construction of recombinant plasmids

The plasmids used in this study are listed in Table 1. The pPGK-CL1 plasmid isolated by yeast functional screening was used for amplifying the *IoPXR1* and *IoGAS1* genes. DNA fragments corresponding to the two coding regions detected in pPGL-CL1 were amplified (separately) by PCR from genomic DNA using the primers listed in Table 2. The pPGK-IoPXR1 plasmid for *IoPXR1* expression in yeast was constructed by inserting the 0.86-kbp *Eco*RI-*Bam*HI DNA fragment containing the *IoPXR1* coding region into the pPGK vector. The pPGK-IoGAS1 plasmid for *IoGAS1* expression in yeast was constructed by inserting the 1.64-kbp *Eco*RI-*Bam*HI DNA fragment spanning the *IoGAS1* coding region into the pPGK vector. Two glutamate residues at position 161 and 262 of *IoGAS1* were replaced with glutamine by performing site-directed mutagenesis using the PrimeSTAR mutagenesis basal kit (Takara Bio). To generate the plasmids pPGK-IoGAS1(E161Q) and pPGK-IoGAS1(E262Q) containing separate missense mutations (E161Q and E262Q, respectively) in the *IoGAS1* gene, the pPGK-IoGAS1 plasmid was used as a template for PCR amplification with the primer pairs listed in Table 2. DNA sequencing confirmed the specific mutagenesis of codon 161 and 262 of the *IoGAS1* gene.

**Table 2:** Primers used for gene cloning and northern blotting.

Gene	Primer sequence (5’ to 3’)
Primers used for gene cloning	
*IoPXR1*	forward: ATGˇAATTCATGGGTCTCGCAGGAACAAG
	reverse: GAGˇGATCCTCATTTGATCATGAAGATTTC
*IoGAS1*	forward: ATGˇAATTCATGAAGTTCTCAAAGTCTCTCGC
	reverse: GAGˇGATCCTCAGATCAAAATCATAGAGATGGAGC
Primers used for northern blotting	
*IoGAS1*	forward: TCCCTTTGTGATTGTGTCGA
	reverse: ATAGAGATGGAGCCGACAAC
Primers used for site-directed mutagenesis	
*IoGAS1* (E161Q)	forward: TAAC**CAA**GTCATTACTAATTCCACAAA
	reverse: GTAATGAC**TTG**GTTACCGGCAAAGAAC
IoGAS1 (E262Q)	forward: TTTTCT**CAA**TATGGTTGTAACGAAGTC
	reverse: ACCATA**TTG**AGAAAAGAAAATCGGAAC

Recognition sites for restriction enzymes are underlined and their cleavage sites are indicated by small arrowheads.

The glutamine codons used to replace the E161 and E262 codons are indicated in bold.

### Yeast transformation

Yeast was transformed with the plasmids pPGK-CL1, pPGK-IoPXR1, pPGK-IoGAS1, pPGK-IoGAS1(E161Q), or pPGK-IoGAS1(E262Q) using a kit for yeast transformation (Yeastmaker^™^ yeast transformation system, Clontech Laboratories, Mountain View, CA, USA). The wild-type *S*. *cerevisiae* strain BY4742 was transformed with pPGK-CL1, pPGK-IoPXR1, or pPGK-IoGAS1 to generate the recombinant strains B4-CL1, B4-IoPXR1, and B4-IoGAS1, respectively. Furthermore, the *S*. *cerevisiae gas1Δ* strain was transformed with pPGK-IoGAS1, pPGK-IoGAS1(E161Q), or pPGK-IoGAS1(E262Q) to construct the recombinant strains D1-IoGAS1, D1-IoGAS1(E161Q), or D1-IoGAS1(E262Q), respectively. The pPGK empty parent vector was transformed into BY4742 and *gas1Δ* to create B4-CON and D1-CON, respectively, for use as control strains. The resulting yeast strains are summarized in Table 1.

### Aerobic growth experiments

B4-CON and B4-IoGAS1 cells were pre-cultivated aerobically in SCD medium for 16 h at 30°C. The cells were then washed with sterile water and inoculated into SCD medium without salt at pH 2.2 and into SCD medium supplemented with 7.5% Na_2_SO_4_ at pH 2.5. The initial absorbance at 600 nm (A_600_) in both cases was around 0.02. During cultivation, cell growth was monitored by A_600_ measurements using a bio-microplate reader (HiTS, Scinics Corporation, Tokyo, Japan). All cultivations in 96-well microplates were performed at 30°C with mild agitation at 150 rpm using the HiTS microplate reader. Cultivation experiments were repeated three times, yielding standard deviations of less than 10%.

### Phylogenetic analysis

The sequences were aligned using the CLUSTAL W (version 1.83) computer program [50]. For phylogenetic analysis, the full-length amino acid sequences of related proteins belonging to the GH72 family of yeasts and filamentous fungi were obtained from the UniProtKB and GenBank databases. The phylogenetic tree was constructed by using two kinds of analytical methods: the neighbor-joining method [51] in the CLUSTAL W program (ver. 1.83) and the two-parameter distance model of Kimura [52]. Bootstrap analysis [53] was performed with 1000 bootstrap replicates for the neighbor-joining tree. The phylogenetic tree was visualized with the TREEVIEW program [54].

### Extraction of RNA and northern blotting

Expression of the endogenous *IoGAS1* gene in *I*. *orientalis* was analyzed by northern hybridization with a probe specific for the *IoGAS1* sequence. The probe used to detect mRNA for the *IoGAS1* gene was amplified by PCR using the primers listed in Table 2. *I*. *orientalis* yeast cells (NBRC1279 strain) grown as described above were harvested at the times indicated in the Results and Discussion section below. Total RNA was extracted using a FastRNA Pro Red kit (MP Biomedicals, Irvine, CA, USA). After DNase I (Takara Bio) treatment, the extracted RNA solution was purified using an RNeasy Mini kit (Qiagen, Hilden, Germany). For northern hybridization, 10 μg of RNA was separated by denaturing electrophoresis on an agarose gel (1.3%) containing 2% formamide, then transferred to a nylon membrane (Biodyne Plus, Pall Corporation, Port Washington, NY, USA). The fixed membrane was hybridized with digoxigenin (DIG) -labeled DNA fragments in DIG Easy Hyb (Roche Diagnostics, Indianapolis, IN, USA) at 68°C for 15 h. The membrane was washed twice with 2× SSC and 0.1% SDS for 10 min at room temperature, and then twice with 0.1× SSC and 0.1% SDS for 15 min at 68°C. DIG-labeled DNA fragments on the washed membrane were detected by alkaline phosphatase-conjugated anti-DIG antibody (Roche) and chemiluminescent detection methods.

### Test of calcofluor white and SDS sensitivity

Calcofluor white (CFW) and sodium dodecyl sulfate (SDS) sensitivities were determined by spotting diluted cultures on plates containing these compounds, as described previously [55]. Briefly, D1-CON, D1-IoGAS1, D1-IoGAS1(E161Q), and D1-IoGAS1(E262Q) cells were incubated in SCD medium overnight at 30°C. The cell density of these growing cultures was standardized with sterile water at an OD_600_ of 0.1, and 2 μL of 3-fold serial dilutions of the four strains (D1-CON, D1-IoGAS1, D1-IoGAS1(E161Q), and D1-IoGAS1(E262Q)) were spotted on SCD containing 10 μg/mL of CFW or 50 μg/mL of SDS, and grown at 30°C for 3 days. In parallel, the cells were also spotted on a control plate (SCD without potential inhibitors), thus allowing comparison with the growth rate of the strains grown on SCD medium containing CFW or SDS after 3 days’ incubation at 30°C.

### Fermentation

For anaerobic batch fermentation using B4-CON and B4-IoGAS1, the recombinant yeast strains were first cultivated aerobically in 5 mL of SCD medium for 36 h at 30°C. Then, the culture was centrifuged at 6000 × g for 5 min at 4°C, and the pelleted cells were washed and resuspended in distilled water. These cells were inoculated into 20 mL of fermentation medium (SCD-2 medium with or without salt (5% Na_2_SO_4_) at pH 2.5 or pH 2.0). For all strains, the initial cell density in the fermentation medium was adjusted to an OD_600_ of approximately 8.3. During fermentation, cell growth was monitored by measuring the absorbance at 600 nm with a UV-2450 spectrophotometer (Shimadzu, Kyoto, Japan). Anaerobic batch fermentations were performed at 30°C in sterile closed bottles (50 mL) with magnetic stirring, as described previously [56]. Samples (0.3 mL) of fermentation broth were removed at specified intervals and diluted four-fold with 8 mM H_2_SO_4_. These diluted samples were stored at -30°C for high-performance liquid chromatography (HPLC) analysis of the substrates and fermentation products. All experiments were performed in triplicate.

### Analysis of substrates and fermentation products

The concentrations of substrates (glucose) and fermentation products (ethanol, glycerol, and acetic acid) were determined using an HPLC apparatus (Jasco, Tokyo, Japan) equipped with a refractive index detector (RI-2031Plus; Jasco), using an Aminex HPX-87H column (Bio-Rad) and a Cation H Refill Guard column (Bio-Rad). The HPLC apparatus was operated at 65°C, with 5 mM H_2_SO_4_ as the mobile phase, at a flow rate of 0.6 mL/min. The injection volume was 20 μL.

## Results and Discussion

### Screening for potential low-pH-tolerance genes from *I*. *orientalis*

The use of yeasts tolerant to acid (low pH) and salt stress is of industrial importance for several bioproduction processes. To identify and isolate novel proteins involved in low-pH tolerance, we screened a genomic DNA library from *I*. *orientalis* NBRC1279 constructed with a multicopy plasmid, pPGK [46], containing the promoter and terminator of the phosphoglycerate kinase (PGK) -encoding locus (see the “Materials and Methods” section). The genomic library was transformed into wild-type *S*. *cerevisiae* strain BY4742, and approximately 8.6 × 10^4^ independent transformants were plated onto SCD plates containing medium adjusted to pH 2.0. Incubation for 20 days at 30°C resulted in the growth of a single colony, denoted B4-CL1; this isolate was characterized in subsequent experiments.

**Fig 1:**
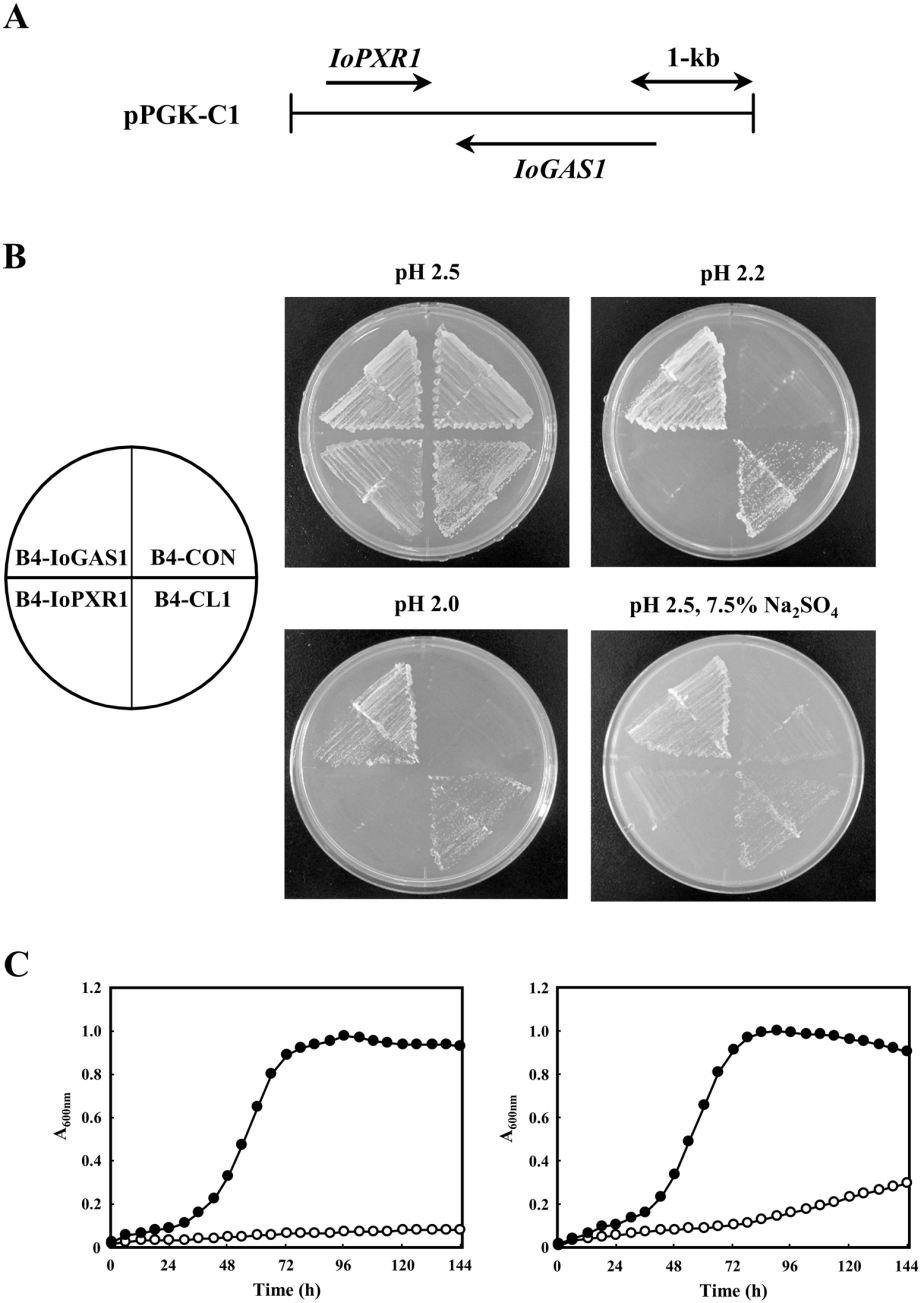
Isolation of *IoGAS1* and demonstration of the gene’s involvement in low-pH- and salt-stress tolerance. (A) The structure of the genomic clone selected by growing on pH 2.0 medium is shown schematically. Plasmid pPGK-C1, isolated in this study, was found to contain the *IoPXR1* and *IoGAS1* genes. Arrows indicate the coding regions as denoted. (B) The recombinant *S*. *cerevisiae* strains B4-CON, B4-CL1, B4-IoPXR1, and B4-IoGAS1 were streaked on an SCD plate without salt at pH 2.5, 2.2, and 2.0, and on pH 2.5 medium containing 7.5% Na_2_SO_4_. The pH 2.5 and 2.2 plates were incubated for 6 days and the SCD plate without salt at pH 2.0 was incubated for 13 days, all at 30°C. (C) The aerobic growth of B4-CON (open circles) and B4-IoGAS1 (closed circles) in liquid SCD medium without salt at pH 2.2 (left panel) and in liquid SCD medium supplemented with 7.5% Na_2_SO_4_ at pH 2.5 (right panel) was tracked over 144 h by measuring the absorbance at 600 nm. Values are the means of three independent experiments.

To identify the genes whose products contribute to low-pH tolerance in *S*. *cerevisiae*, the plasmid was isolated from the putative positive yeast clone (B4-CL1 strain). Analyses involving restriction endonuclease digestion and sequencing of the insert in the clone revealed that the insert consisted of 3819 bp and contained two uninterrupted putative ORFs consisting of 849 bp (ORF-1) and 1629 bp (ORF-2) (Fig 1A). The predicted amino acid sequences indicated that the ORF-1 and ORF-2 gene products consisted of 282 and 542 amino acids, respectively. The sequences of these ORFs were compared with GenBank database entries to determine putative functions. The predicted ORF-1 protein showed 62% identity to *S*. *cerevisiae* PXR1, an essential protein involved in rRNA and snoRNA maturation and in the negative regulation of telomerase [57–59]. The longer predicted ORF-2 protein displayed 58–60% identity with both Gas1 of *S*. *cerevisiae* [44,45], which is necessary for cell wall construction, and the Phr1 and Phr2 proteins (known pH-regulated enzymes) of the pathogenic fungus *Candida albicans* [60,61], which are involved in cell wall assembly and required for virulence in that species. Gas1 is an extracellular glycoprotein that is anchored to the cell membrane through a GPI tail; Gas1 exhibits β-1,3-glucanosyltransferase activity, and catalyzes a transglycosylation crosslinking reaction with β-1,3-glucan as a substrate [45]. Based on these homology results, the two identified *I*. *orientalis* genes encoding ORF-1 and ORF-2 were designated as *IoPXR1* and *IoGAS1*, respectively (Fig 1A).

### Overexpression of the *IoGAS1* gene increases yeast tolerance to acidity and salinity

To determine which of these ORFs is involved in low-pH tolerance, the PCR products of *IoPXR1* and *IoGAS1* were (separately) subcloned into the pPGK vector to create the plasmids pPGK-IoPXR1 and pPGK-IoGAS1, respectively. These plasmids were then introduced into BY4742 to generate strains B4-IoPXR1 and B4-IoGAS1, respectively. The acid tolerance of these transformants, together with a positive clone (B4-CL1) and a control strain (B4-CON), were tested using the streak test on SCD plates at pH 2.0–2.5 and containing 0–7.5% Na_2_SO_4_ (Fig 1B). All recombinant strains grew on the pH 2.5 SCD plates (Fig 1B, upper left panel). However, B4-CON and B4-IoPXR1 were unable to grow on pH 2.2 medium (Fig 1B, upper right panel), whereas B4-CL1 and B4-IoGAS1 (both of which carry the *IoGAS1* gene) were able to grow at pH 2.2 (Fig 1B, upper right panel) and at pH 2.0 (Fig 1B, lower left panel). These results suggested that overexpression of *IoGAS1* in *S*. *cerevisiae* confers low-pH tolerance. B4-CL1 and B4-IoGAS1 also grew on pH 2.5 plates containing 7.5% Na_2_SO_4_, whereas B4-CON and B4-IoPXR1 did not (Fig 1B, lower right panel). These results suggested that overexpression of *IoGAS1* renders yeast cells tolerant not only to low pH, but also to high salt concentrations. Furthermore, B4-IoGAS1 grew better than B4-CL1 on these acid and/or salt plates (Fig 1B), a difference that is attributed to the differing promoter strengths in the two genetic constructs.

Next, the tolerance to low pH and salt by the stress-tolerant B4-IoGAS1 strain and a control strain (B4-CON) was examined in acidic liquid medium (SCD) with and without a high concentration of Na_2_SO_4_ (Fig 1C) under aerobic conditions. In SCD medium at pH 2.2 with no salt, B4-IoGAS1 grew during the initial 72 h of cultivation (0–72 h; Fig 1C, left panel), but showed little growth thereafter (72–144 h; Fig 1C, left panel). In contrast, B4-CON showed little growth throughout the entire 144-h incubation period (Fig 1C, left panel), consistent with the lack of growth observed in Fig 1B. On SCD medium at pH 2.5 and containing 7.5% Na_2_SO_4_, B4-IoGAS1 also grew at the early stage of cultivation (0–96 h; Fig 1C, right panel), with a gradual decrease in growth thereafter (96–144 h; Fig 1C, right panel). In contrast, B4-CON showed poor growth under the same conditions (Fig 1C, right panel), consistent with the growth pattern seen in Fig 1B. These results strongly suggested that expression of *IoGAS1* is required for the growth of yeast at low pH and high salt concentrations.

### Identification of the *IoGAS1* gene product as a putative GPI-anchored protein

As mentioned above, the 1629-bp nucleotide sequence of the *IoGAS1* ORF was predicted to encode a 542-amino-acid protein. NCBI BLAST analysis of *IoGAS1* revealed that the best matches to known proteins in the database were to *S*. *cerevisiae* Gas1p (60% identity) and to *C*. *albicans* Phr1p (58% identity) and Phr2p (59% identity) (referred to as ScGas1p, CaPhr1p, and CaPhr2p, respectively) [60–62]. All three proteins (ScGas1p, CaPhr1p, and CaPhr2p) are believed to be major glycoproteins localized to the plasma membrane through a GPI anchor and to be important for the maintenance of cell wall integrity [60,63–65]. CaPhr1p and CaPhr2p have been shown to process β-glucans in the cell wall and are required for proper cross-linking of 1,3-β and 1,6-β glucans [66].

**Fig 2:**
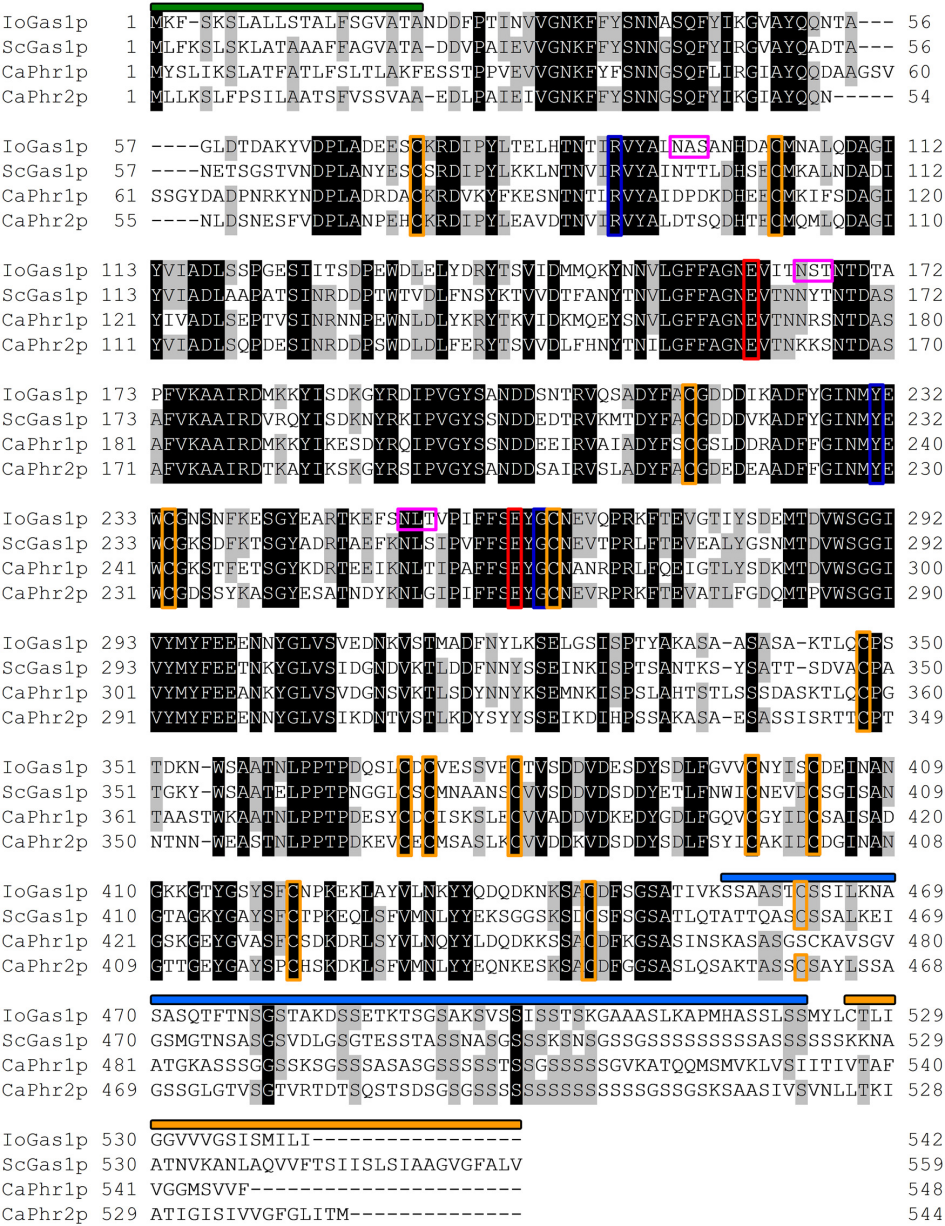
Comparison of the predicted amino acid sequences of the *IoGAS1*, *ScGAS1*, *CaPHR1*, and *CaPHR2* products (IoGAS1p, ScGAS1p, CaPHR1p, and CaPHR2p, respectively). Identical and conserved amino acids are shaded in black and grey, respectively. *Io*, *Issatchenkia orientalis*; *Sc*, *Saccharomyces cerevisiae*; *Ca*, *Candida albicans*. Conserved sequence motifs that were identified in the homology model are annotated as follows: green overlining, signal peptide; orange overlining, transmembrane helices; blue overlining, serine-rich region. Functionally conserved residues are indicated as follows: red boxes, catalytic glutamate residues; blue boxes, substrate recognition sites; orange boxes, cysteine residues that may participate in disulfide bonds; pink boxes, putative *N*-glycosylation motifs.

The predicted structural features and the overall identity of the putative IoGas1 protein, and of the homologous ScGas1p, CaPhr1p, and CaPhr2p proteins, are shown in Fig 2. The four proteins exhibited similarity along their entire lengths; the N- and C-terminal hydrophobic regions typical of GPI-anchored proteins, together with several other functionally significant features, were conserved. As with ScGas1p, CaPhr1p, and CaPhr2p, the first 21 amino acids of the predicted full-length IoGas1p constitute a predicted signal peptide, and the hydrophobic carboxyl terminus is predicted to form transmembrane helices [67] that are implicated in GPI anchoring. All the strictly conserved residues of ScGas1p, CaPhr1p, and CaPhr2p are also present in the putative catalytic domain of IoGas1p [68]; in particular, in the A-G-N-**E**-V and S-**E**-Y-G-C motifs, the two glutamic residues (E161 and E262, in bold) that are essential for enzymatic activity of β-1,3-glucanosyltransferase [69,70] are conserved. These residues have been proposed to serve as a proton donor and nucleophile, respectively [69]. Moreover, the residues corresponding to R90, Y231, and G264 of ScGas1p, residues that might be important for substrate recognition and orientation/activation of the nucleophilic glutamic catalytic residue [68], are also conserved in IoGas1p. In IoGas1p, three putative *N*-glycosylation motifs are detected, at amino acid positions N95 (the amino acids in the *N*-glycosylation consensus sequence NAS), N165 (NST), and N253 (NLT) (Fig 2). IoGas1p also contains a serine-rich region in which 24 serines are clustered in a region between residues S456 and S522 (Fig 2). The serine-rich region in ScGas1p is a target for *O*-glycosylation and is dispensable for activity [45, 71]. In addition, the positions of 14 of the 15 cysteine residues of IoGas1p (positions 74, 103, 216, 234, 265, 348, 370, 372, 379, 398, 403, 421, 445, and 462) that might be involved in intra-/interdomain disulfide bonds [70] are conserved among these proteins (Fig 2). Previous work in ScGas1p has shown that C74 is necessary for the correct folding of the protein, whereas C103 and C265 are dispensable [70].

**Fig 3:**
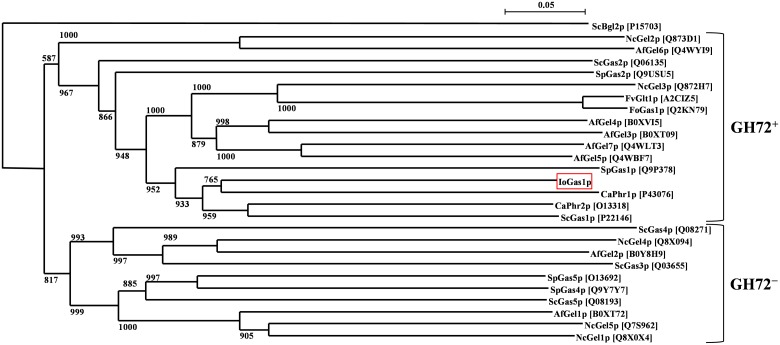
The neighbor-joining tree obtained using CLUSTAL W ver. 1.83, indicating the phylogenetic relationships among the putative β-1,3-glucanosyltransferases belonging to the GH72 family. This phylogenetic tree was constructed using the full-length amino acid sequences obtained from UniProtKB and GenBank databases. UniProtKB/Swiss-Prot accession numbers are indicated in brackets. Branch lengths are proportional to sequence divergence [76]. The numbers shown are bootstrap values. The sequences from *S*. *cerevisiae* are: ScGas1p (UniProtKB/Swiss-Prot accession number P22146), ScGas2p (Q06135), ScGas3p (Q03655), ScGas4p (Q08271), ScGas5p (Q08193); from *C*. *albicans*: CaPhr1p (P43076), CaPhr2p (O13318); from *Aspergillus fumigatus*: AfGel1p (B0XT72), AfGel2p (B0Y8H9), AfGel3p (B0XT09), AfGel4p (B0XVI5), AfGel5p (Q4WBF7), AfGel6p (Q4WYI9), AfGel7p (Q4WLT3); from *Schizosaccharomyces pombe*: SpGas1p (Q9P378), SpGas2p (Q9USU5), SpGas4p (Q9Y7Y7), SpGas5p (O13692); from *Neurospora crassa*: NcGel1p (Q8X0X4), NcGel2p (Q873D1), NcGel3p (Q872H7), NcGel4p (Q8X094), NcGel5p (Q7S962); from *Fusarium oxysporum*: FoGas1p (Q2KN79); and from *Fusarium verticilliodes*: FvGlt1 (A2CIZ5). The *S*. *cerevisiae* β-1,3-endoglucanase, ScBgl2p (P15703), was used as the outlier reference sequence.

The ScGas1p homologues belong to a family of glycosyl hydrolases (family GH72), a widely conserved group of yeast and fungal enzymes that are involved in cell wall assembly [72]. Based on the presence or absence of the approximately 100 residue C-terminal cysteine-rich domain, named the Cys-box, the GH72 family can be divided into the GH72^+^ and GH72^-^ subfamilies, respectively [43], and these two subfamilies are also evident in the phylogenetic tree (Fig 3). Most fungi contain multiple members of GH72 family of enzymes [73]. For example, *S*. *cerevisiae* contains four other proteins (ScGas2p–ScGas5p) that are paralogues of ScGas1p [45], and two (ScGas1 and ScGas2) belong to the GH72^+^ subfamily [43]. *C*. *albicans* contains five GH72 enzymes, of which CaPhr1p and CaPhr2p belong to the GH72^+^ subfamily. ScGas1p, CaPhr1p, and CaPhr2p are homologous to *Aspergillus fumigatus* Gel1p, a β-1,3-glucanosyltransferase isolated from the cell wall of this fungal pathogen [74]. *A*. *fumigatus* contains seven Gel family members (AfGel1p–AfGel7p), of which only AfGel1p and AfGel2p belong to the GH72^-^ subfamily; among these seven family members, AfGel4p (a member of the GH72^+^ subfamily) has been demonstrated to be an essential protein [75].

To gain statistical insights into the relationships among these GH72 enzymes, as well as ScGas1p homologues in *Schizosaccharomyces pombe* and *Fusarium oxysporum*, and AfGel1p homologues in *Neurospora crassa*, a phylogenetic tree was constructed using the neighbor-joining method with bootstrap values (n = 1000) in the CLUSTAL W (ver. 1.83) program [50]. The results showed that IoGas1p belongs to subfamily GH72^+^ (Fig 3). This tree also clearly revealed very close relationships between IoGas1p and other members of the GH72^+^ subfamily, including ScGas1p, CaPhr1p, and CaPhr2p (Fig 3). Thus, the nucleotide sequence of *IoGAS1* encodes a 542-aa polypeptide sharing features of the other proteins comprising family GH72.

### *IoGAS1* expression is regulated by environmental pH

**Fig 4:**
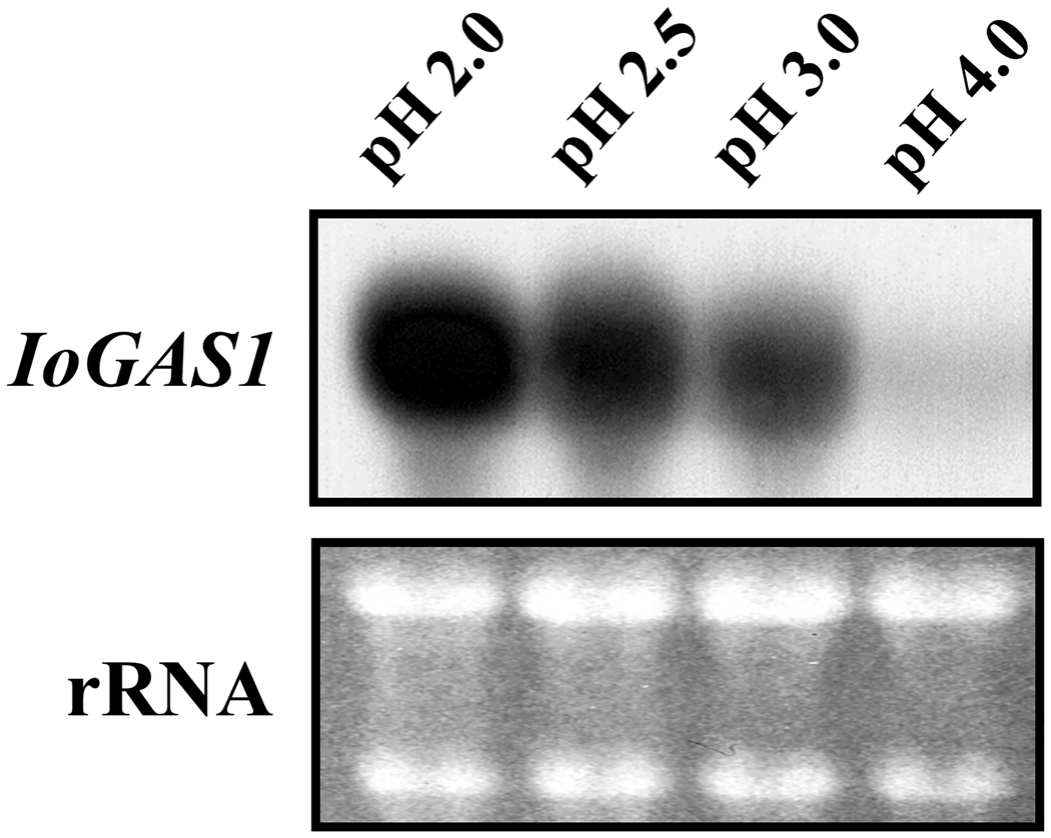
Northern blot hybridization analyses of the *IoGAS1* transcript from *I*. *orientalis* strain NBRC1279. RNA was isolated from yeast cells grown in YPD medium at pH 2.0, pH 2.5, pH 3.0, or pH 4.0, followed by hybridization with a probe specific for the *IoGAS1* coding sequence. Ethidium bromide-stained rRNA bands are shown as loading controls.

It has been shown that different GH72 paralogues are expressed at specific stages of the yeast life cycle. In *S*. *cerevisiae*, *GAS1* and *GAS5* are expressed mainly during the vegetative growth phase, whereas the expression of *GAS2* and *GAS4* is repressed during vegetative growth and is activated during the sporulation phase [77,78]. *S*. *cerevisiae* is thus not capable of pH-regulated dimorphism, whereas in *C*. *albicans*, *PHR1* and *PHR2* display a unique complementary pH-dependent expression pattern. Expression of *PHR1* is detected only when the ambient pH is above 5.5, and increases at more alkaline pH values [60]. In contrast, *PHR2* exhibits the inverse pattern, being repressed at pH values above 6 and progressively induced at more acidic pH values, with maximum expression at pH 4 to 5 [61]. Interestingly, the pH optima of the β-1,3-glucanosyltransferases are 5.8 for Phr1p and 3 for Phr2p [79], an observation that is consistent with the pH-regulated expression of the corresponding genes, whereas *S*. *cerevisiae* Gas1 protein has a moderately acidic pH optimum for activity (3.5–4.5) [80]. In light of the considerable homology of *IoGAS1* to *PHR1* and *PHR2*, we further evaluated whether endogenous expression of this *I*. *orientalis* gene was similarly responsive to changes in the ambient pH. Northern hybridization analyses showed that this pH-dependence was indeed the case, as demonstrated below (Fig 4). To this end, *I*. *orientalis* NBRC1279 was grown anaerobically in YPD medium adjusted to different acidic pH values (4.0, 3.0, 2.5, and 2.0), total RNA was prepared, and the transcript of the *IoGAS1* gene under the control of its natural promoter was investigated.

Northern hybridization analyses with a probe specific for the *IoGAS1* coding sequence detected a single band of approximately 2 kb, and the intensity of this band varied with the pH of the fermentation medium (Fig 4). Incremental increases in *IoGAS1* mRNA were seen in the pH range from 4.0 to 2.0. High levels of expression of *IoGAS1* were detected with the *IoGAS1* probe when cells were fermented at pH 2.0. In contrast, very little or no expression was detected when cells were fermented at pH 4.0. Thus, in *I*. *orientalis*, *IoGAS1* expression was repressed at pH 4.0 but induced at more acidic pH, indicating that transcription of the *IoGAS1* gene is pH-dependent. Therefore, *IoGAS1* is differentially regulated in response to the pH of the growth medium. This pH-dependent expression pattern of *IoGAS1* is unique among the reported members of the *GAS*/*PHR* gene family, although it is somewhat similar to that of *C*. *albicans PHR2*, in which more moderately acidic pH values (pH 4–5) of the medium are required for maximum expression. These patterns may correlate with the low-pH adaptation and acid tolerance response of *I*. *orientalis* and imply that the cellular function performed by *IoGAS1* is required at acidic pH. In any case, this finding clearly suggests that *I*. *orientalis* possesses a novel pH-regulated system, presumably enabling the organism to adapt to environments with diverse pH values. To our knowledge, this represents the first description of an environmentally regulated gene in the multi-stress-tolerant yeast *I*. *orientalis*.

### Overexpression of *IoGAS1* complements *GAS1* deletion in *S*. *cerevisiae*

**Fig 5:**
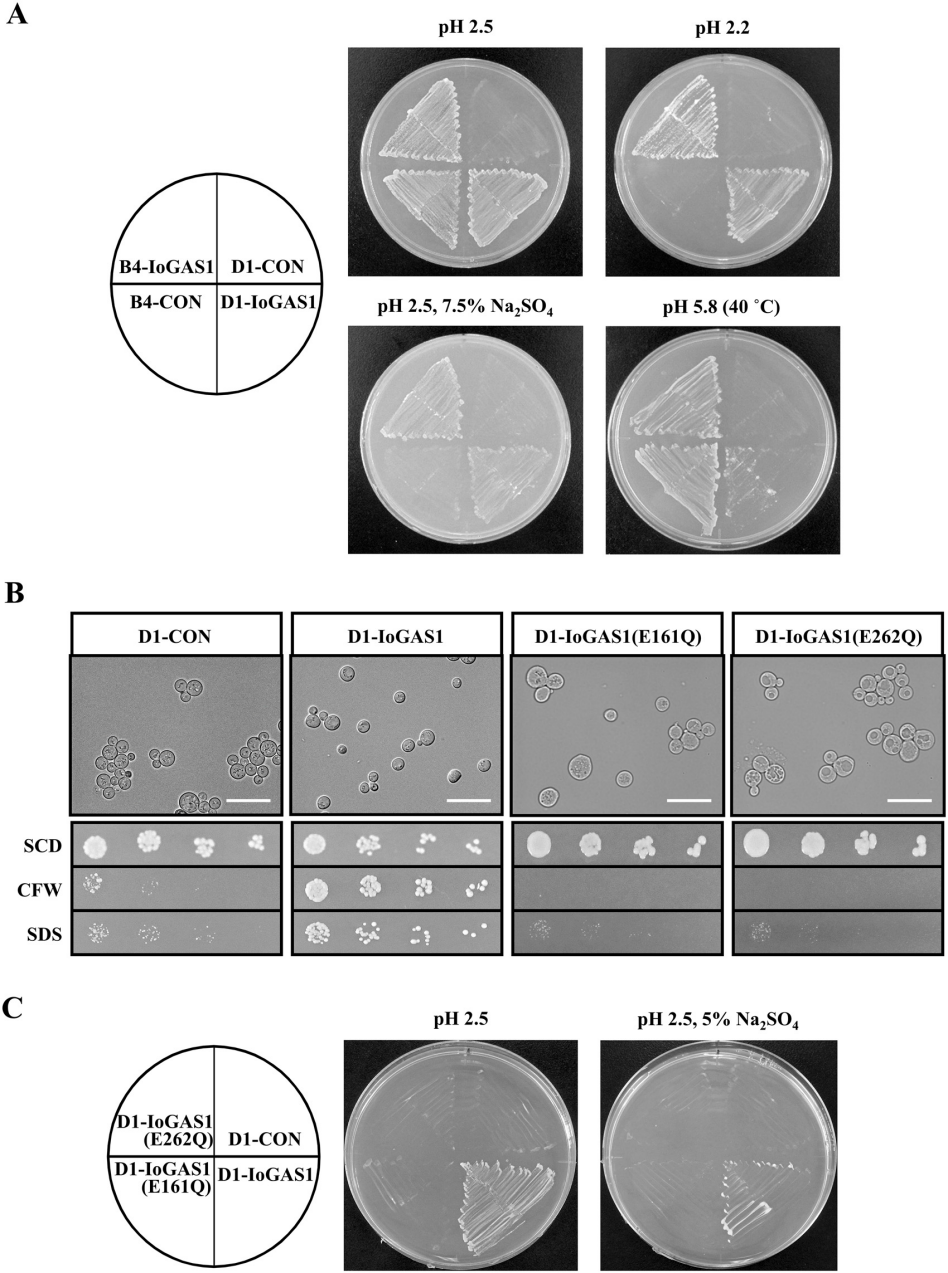
Analysis of complementation of the *S*. *cerevisiae gas1* knockout mutant phenotype by the expressed *IoGAS1* gene. (A) The recombinant *S*. *cerevisiae* strains D1-CON, D1-IoGAS1, B4-CON, and B4-IoGAS1 were streaked on an SCD plate without salt at pH 2.5 or pH 2.2, or on an SCD plate at pH 2.5 containing 7.5% Na_2_SO_4_, followed by incubation at 30°C for 3 days, 6 days, or 6 days, respectively. In addition, these yeast strains were grown on an SCD plate without salt at pH 5.8 at 40°C for 8 days. (B) The cell morphology of D1-CON, D1-IoGAS1, D1-IoGAS1(E161Q), and D1-IoGAS1(E262Q) was observed by microscopy during the stationary phase of growth in SCD medium at pH 4.0 (upper panels). Each white bar denotes 20 μm. Calcofluor white (CFW) and SDS sensitivity of D1-CON, D1-IoGAS1, D1-IoGAS1(E161Q), and D1-IoGAS1(E262Q) (lower panels): Aliquots (2 μL) of 3-fold serial dilutions of the four strains (D1-CON, D1-IoGAS1, D1-IoGAS1(E161Q), and D1-IoGAS1(E262Q)), starting at OD (600 nm) = 0.1, were spotted onto SCD plates, SCD plates supplemented with 10 μg/mL of CFW, or SCD plates supplemented with 50 μg/mL of SDS, and grown at 30°C for 3 days. (C) The recombinant *S*. *cerevisiae* strains D1-CON, D1-IoGAS1, D1-IoGAS1(E161Q), and D1-IoGAS1(E262Q) were streaked on an SCD plate without salt at pH 2.5 or on an SCD plate at pH 2.5 containing 5% Na_2_SO_4_, followed by incubation at 30°C for 3 days or 6 days, respectively.

The above finding regarding the pH-dependent transcriptional regulation of *I*. *orientalis IoGAS1*, in addition to the sequence similarities among IoGas1p, *S*. *cerevisiae* Gas1p, and *C*. *albicans* Phr1p and Phr2p, suggested that the IoGas1 protein may be a functional homologue of the ScGas1, CaPhr1, and CaPhr2 proteins. Furthermore, both *CaPHR1* and *CaPHR2* complement a *gas1Δ* deletion in *S*. *cerevisiae* [63], indicating that these homologues are not only structurally but also functionally similar. Based on these findings, we tested the ability of *IoGAS1* to functionally replace *S*. *cerevisiae GAS1*, particularly with regard to the growth and morphological aberrations of the *S*. *cerevisiae gas1Δ* null mutant. For this purpose, the *IoGAS1* gene was transformed into the *gas1Δ* strain to generate D1-IoGAS1. D1-IoGAS1, and a control strain, D1-CON, together with B4-CON and B4-IoGAS1 in the wild-type BY4742 background, were then compared in terms of aerobic growth levels on SCD plates at different pH values with or without salt (Fig 5A). Interestingly, D1-CON, in which *GAS1* was deleted, exhibited very limited growth on an SCD plate at pH 2.5 (Fig 5A, upper left panel). On the other hand, the other three recombinant strains (D1-IoGAS1, B4-CON, and B4-IoGAS1) grew reasonably well on the plate, indicating that the *IoGAS1* gene can complement the growth defects due to a *gas1* null mutation in *S*. *cerevisiae*. This complementation was even more evident under more strongly acidic conditions (pH 2.2): not only D1-CON, but also B4-CON was unable to grow on SCD plates at pH 2.2, whereas both D1-IoGAS1 and B4-IoGAS1, carrying the *IoGAS1* gene, grew at pH 2.2 (Fig 5A, upper right panel). A similar result was obtained for the growth condition of pH 2.5 and 7.5% Na_2_SO_4_ (Fig 5A, lower left panel), demonstrating that IoGas1p is functionally homologous to ScGas1p.

It was previously reported from a genome-wide analysis that deletion of *GAS1* in *S*. *cerevisiae* resulted in reduced growth at 37°C [81]. This result suggests that the *gas1* mutant is sensitive to heat stress as well as low-pH and salt stress, although currently no relationship is known in yeast between β-1,3-glucanosyltransferases and thermotolerance. To test the ability of *IoGAS1* to complement the high-temperature growth defect of the *S*. *cerevisiae gas1Δ* mutation, four yeast strains (D1-CON, D1-IoGAS1, B4-CON, and B4-IoGAS1) were compared for their growth ability at 40°C and normal pH (pH 5.8) (Fig 5A, lower right panel). After 8 days’ incubation, B4-CON and B4-IoGAS1 in the wild-type background showed good growth at high temperature, while D1-CON, which lacks *IoGAS1*, was unable to grow. D1-IoGAS1 also grew at high temperature, but its growth was considerably weaker than that of the wild-type background strains (B4-CON and B4-IoGAS1). Thus, the *IoGAS1* gene can partially complement the slow-growth-rate defect of the *gas1Δ* strain in SCD medium at high temperature (40°C).

We then examined whether *IoGAS1* can complement the morphological defect of the *S*. *cerevisiae gas1Δ* mutation. It has been reported that *S*. *cerevisiae* (both haploid and diploid) *gas1* null mutants have a rounded shape, larger cell diameter, and multibudded phenotype compared with wild-type cells, especially in stationary phase [44,62]. In addition to the abnormal morphology of the *gas1Δ* mutant, mutations of the *GAS1* gene lead to a loss of 1,3-β-glucan and an increase in the levels of chitin and mannan in the fungal cell wall [64,65,82]. Consequently, the morphological features of D1-CON and D1-IoGAS1 during stationary phase were investigated by microscopic analysis (Fig 5B, upper left half panels). As expected, D1-CON cells lacking *GAS1* exhibited morphological aberrations, becoming rounded and enlarged, highly vacuolated, and often carrying more than one bud (Fig 5B, upper leftmost panel). Notably, the *gas1Δ* cells showed the typical phenotype of two buds attached to the mother cell (a Mickey Mouse-like appearance), a finding that is consistent with previous reports [83,84]. In contrast, D1-IoGAS1 expressing *IoGAS1* exhibited normal yeast morphology (Fig 5B, upper second panel from left). Thus, this microscopic analysis revealed that *IoGAS1* is able to complement the morphological defects of the *gas1Δ* mutant, restoring the normal morphology in stationary phase.

As mentioned above, *GAS*-class genes are implicated in cell wall generation, cross-linking, and stability [45]. The cell wall of the *gas1* null mutant is highly resistant to zymolyase (β-1,3-glucanase); more sensitive to calcofluor white (CFW), a cell wall-perturbing agent that interferes with cell wall assembly; and more sensitive to sodium dodecyl sulphate (SDS), a potent osmotic destabilizing agent [63,85]. These observations suggested that the *S*. *cerevisiae gas1Δ* mutant strain exhibits increased permeability, presumably due to weakening of the cell wall. Accordingly, we used spot assays to evaluate the ability of *IoGAS1* to complement the growth defect of the *gas1Δ* strain in the presence of CFW or SDS. As anticipated, growth of the *GAS1*-deficient *S*. *cerevisiae* strain D1-CON on solid medium is impaired in the presence of 10 μg/mL CFW or 50 μg/mL (Fig 5B, lower leftmost panels). In contrast, transformation of the *S*. *cerevisiae gas1Δ* strain with the *IoGAS1* gene (D1-IoGAS1) yielded a strain that grew normally on solid medium containing CFW or SDS (Fig 5B, lower second panels from left). Thus, *IoGAS1* fully complemented the CFW and SDS sensitivity of *gas1* mutant cells. The finding that introduction of the *IoGAS1* gene into the *S*. *cerevisiae gas1Δ* strain reverses the mutant phenotypes strongly suggests that *IoGAS1* and its homologue *ScGAS1* have similar roles in maintaining yeast cell wall integrity. In addition, these results demonstrate that a novel cell wall-related protein is directly involved in yeast tolerance to acidic and salinity stress.

### Essential role of the strictly conserved catalytic residues E161 and E262 in IoGas1p

*S*. *cerevisiae* Gas1p, as well as *C*. *albicans* Phr1p and Phr2p, have been shown to exhibit in vitro β-1,3-glucanosyltransferase activity, an activity that plays an important role in β-1,3-glucan remodeling processes during yeast cell wall synthesis [74]. As shown in Fig 2, two glutamic residues (E161 and E262) of IoGas1p can be aligned with corresponding residues in *S*. *cerevisiae* Gas1p, *C*. *albicans* Phr1p, and *C*. *albicans* Phr2p. In these other proteins and *A*. *fumigatus* Gel1p, these strictly conserved Glu residues are believed to form part of the catalytic domains of these enzymes; site-specific mutation of these Glu residues abolishes enzyme activity and/or the ability to complement cells mutated in the corresponding genes [43,66,69,70]. Importantly, *S*. *cerevisiae* Gas1p with glutamine substitutions at these two catalytic residues remains structurally intact, but the enzymatic activity of such a mutant protein is completely ablated [70]. In our work, we sought to determine whether E161 and E262 correspond to the essential catalytic residues of the *Saccharomyces* and *Candida* homologues, specifically by assessing whether these residues in IoGas1p are required for the ability of IoGas1p to rescue the morphological defects of the *S*. *cerevisiae gas1Δ* mutant. Therefore, we used site-specific mutagenesis to generate *IoGAS1* plasmids encoding proteins in which E161 and E262 were (separately) replaced with glutamine. These mutated genes were transformed into the *gas1Δ* strain to create D1-IoGAS1(E161Q) and D1-IoGAS1(E262Q), respectively. Microscopic observation of these cells showed that D1-IoGAS1(E161Q) and D1-IoGAS1(E262Q) cells exhibited abnormal morphologies (Fig 5B, upper right two panels), like D1-CON cells lacking *GAS1*. Thus, neither the E161 nor the E262 mutant allele was able to complement the morphological defect of the *gas1Δ* mutant. This result is consistent with a previous finding, in *C*. *albicans*, showing that analogous missense mutations of *PHR1* (yielding substitutions at protein residues E169 or E270, positions essential for Phr1p enzyme activity) prevented complementation of the morphological defects of a *phr1Δ* mutant [66]. In addition, these mutant *IoGAS1* constructs were unable to complement the CFW and SDS sensitivities of *S*. *cerevisiae gas1* mutant cells (Fig 5B, right half of the lower panels). We infer that these two conserved glutamate residues (E161 and E262) in IoGas1p are required for complementation in *S*. *cerevisiae*, and that IoGas1p and *S*. *cerevisiae* Gas1p possess similar enzymatic functions. Thus, as predicted from the alignment with GH72 enzymes, E161 and E262 appear to be active-site residues of IoGas1p that are essential for IoGas1p activity.

Given the above observations, we presumed that the loss of IoGas1p enzymatic activity would affect *S*. *cerevisiae* stress survival and tolerance to low pH and salt. To confirm this prediction, D1-IoGAS1(E161Q) and D1-IoGAS1(E262Q) were examined using the streak test on pH 2.5 plates with and without 5% Na_2_SO_4_. As expected, we found that, D1-IoGAS1(E161Q) and D1-IoGAS1(E262Q) (like D1-CON) were unable to grow on pH 2.5 medium even in the absence of Na_2_SO_4_ (Fig 5C), demonstrating that Glu-161 and Glu-262 in the active site of IoGas1p are necessary for tolerance to low-pH and salt stresses. Considered together, these observations suggest that IoGas1p (like other members of the GH72 family) possesses β-1,3-glucanosyltransferase activity, and that this enzyme activity is directly responsible for acid- and saline-stress tolerance.

At present, the precise mechanisms by which the *IoGAS1* gene regulates low-pH and salt tolerance remain unclear; however, previous studies provide some clues to the gene’s functional properties. First, sphingolipids have been shown to be required for growth at low pH and are essential for a normal rate of transport of GPI-anchored proteins, including Gas1p, from the endoplasmic reticulum (ER) to the Golgi apparatus [86]. Therefore, one explanation for increased survival at low pH is that the rate of transport of IoGas1 protein toward the Golgi apparatus is increased in cells overexpressing IoGas1 in the presence of sphingolipids, leading to a more protective cell wall. It is also possible that IoGas1p is directly involved in the cell wall integrity (CWI) pathway that mediates tolerance to low pH. Low-pH conditions activate the CWI pathway via the stress sensor Mid2p, leading to the phosphorylation of MAP kinase Slt2p/Mpk1p and finally to activation of the transcription factor Rlm1p [87]. IoGas1p may mediate transduction of the signal from the Mid2p sensor to Slt2p/Mpk1p. Interestingly, *GAS1* gene has been shown to genetically interact with both *SLT2* and *RLM1* in *S*. *cerevisiae* [88]. Intriguingly, the β-1,3-glucanosyltransferase activity of Gas1p also has been shown to be involved in the transcriptional silencing [89,90]. In addition, dysfunction of Gas1p has been shown to enhance rRNA integrity by decreasing the activity of cAMP-dependent protein kinase (PKA) in a Slt2p/Mpk1p-dependent manner, thereby inducing the translocalization of Msn2/4p into the nucleus [90]; this result suggests that Gas1p controls PKA activity through Slt2p/Mpk1p. It should also be noted that *GAS1* has critical genetic interactions with genes encoding the histone H3 lysine acetyltransferases Gcn5p and Sas3p, demonstrating that Gas1p plays an important role in chromatin dynamics [91]. These roles of Gas1p are separable from its cell wall function. The mechanism(s) whereby Gas1p can contribute to multiple pathways (including stress tolerance, transcriptional silencing, and chromatin dynamics) remain unknown. However, based on these and other previous observations, we postulate that IoGas1p is directly involved in tolerance to low pH and high salt concentrations through CWI signaling, perhaps by regulating Slt2p/Mpk1p. Further studies will be needed to clarify the role of IoGas1p in relation to stress tolerance and pH regulation.

### Improvement of ethanol fermentation by an *IoGAS1*-overexpressing strain under acidic and high-salt conditions

**Fig 6:**
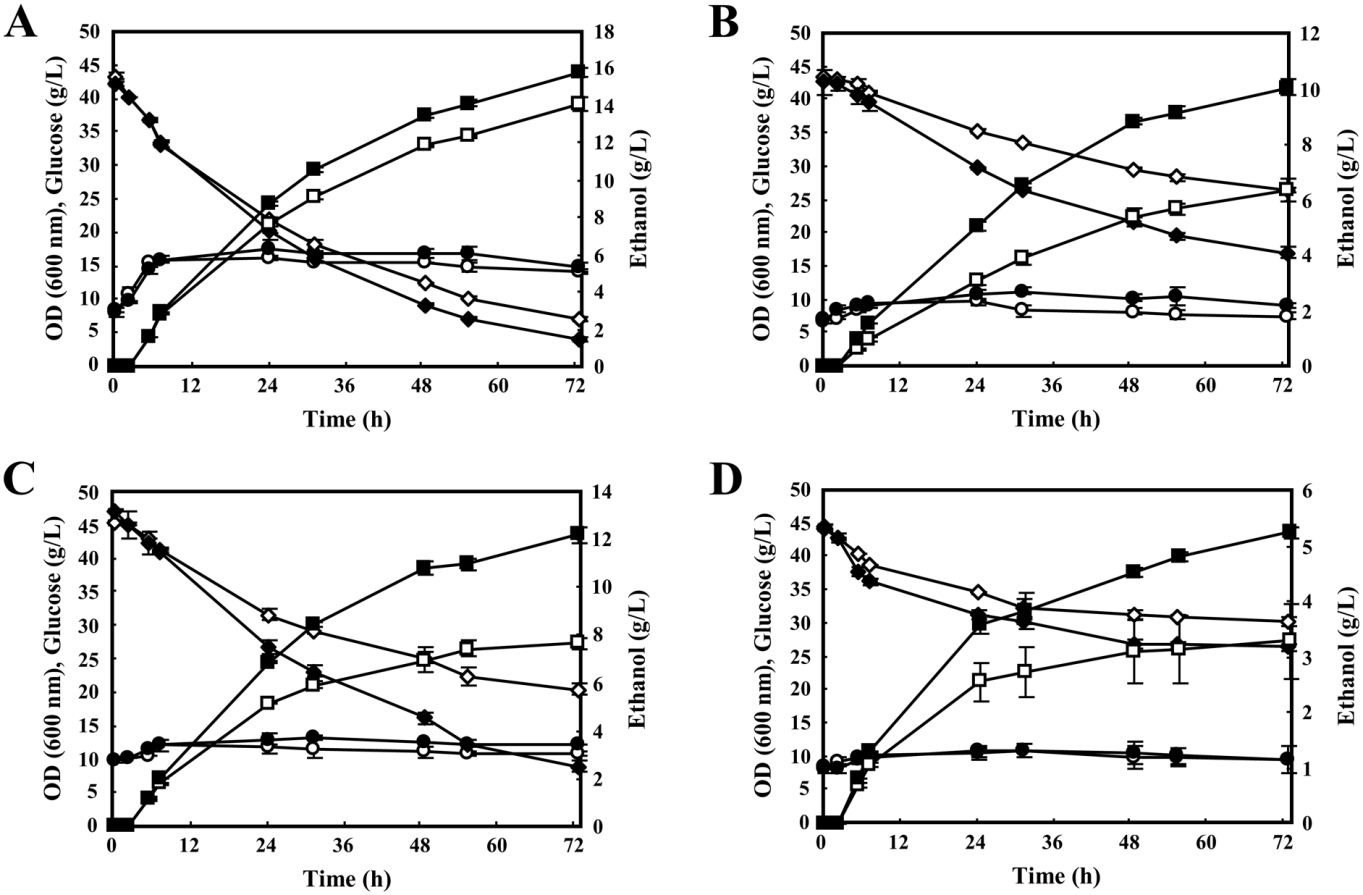
Time-dependent batch fermentation profiles of recombinant yeast strains. *S*. *cerevisiae* B4-CON (open symbols) and B4-IoGAS1 (closed symbols) were grown in SCD-2 medium without the addition of salt (A and B) or with 5% Na_2_SO_4_ (C and D) at pH 2.5 (A and C) or pH 2.0 (B and D) under anaerobic conditions. The OD at 600 nm (circles), concentrations of glucose (diamonds), and concentrations of ethanol (squares) during batch fermentation were measured over 72 h. Glycerol and acetic acid were detected at low levels (data not shown; see text). Values are presented as the means ± standard deviations from three independent experiments.

Finally, we examined whether an *IoGAS1*-overexpressing *S*. *cerevisiae* strain (B4-IoGAS1), which exhibits increased tolerance to extremely low pH and high concentrations of salt, can indeed efficiently ferment sugar to ethanol under low-pH and high-salt-concentration conditions. Glucose (used as the sole carbon source) was fermented using B4-IoGAS1or the control strain B4-CON in medium (SCD-2) at different pH values (pH 2.5 or 2.0) with or without a high concentration (5%) of Na_2_SO_4_ (Fig 6). In the fermentation conducted at pH 2.5 without additional salt, both B4-CON and B4-IoGAS1 grew (as measured by OD_600_) in a linear manner in the early phase of the glucose consumption (0 to 7.5 h) before growth effectively plateaued (Fig 6A). Similar growth (OD_600_) time courses were observed for both strains under each of the tested pH and salt conditions.

In the fermentation conducted at pH 2.5 without additional salt, B4-CON and B4-IoGAS1 consumed 36.2 ± 0.7 and 38.0 ± 0.5 g/L of the glucose, respectively, by 72 h, with ethanol accumulating to 14.1 ± 0.4 and 15.8 ± 0.3 g/L, respectively, by the final time point (Fig 6A). These values corresponded to glucose consumption rates for B4-CON and B4-IoGAS1 of 0.503 ± 0.010 and 0.528 ± 0.008 g/L/h, respectively, and to ethanol production rates of 0.196 ± 0.005 and 0.219 ± 0.004 g/L/h, respectively. Thus, B4-IoGAS1 exhibited nominally elevated parameters, with a 5%-higher glucose consumption rate and a 12%-higher ethanol production rate compared to those of B4-CON. Glycerol and acetic acid were produced as by-products by B4-CON (less than 1.78 g/L and 0.92 g/L, respectively) and B4-IoGAS1 (less than 1.42 g/L and 0.80 g/L, respectively). The ethanol yield of B4-IoGAS1 (0.42 ± 0.00 g of ethanol per gram of consumed glucose) was comparable to that of B4-CON (0.39 ± 0.02 g/g) after 72 h of fermentation.

The difference in the fermentative capacity of B4-CON and B4-IoGAS1 became more evident under more acidic conditions (pH 2.0) (Fig 6B). At pH 2.0 without the addition of salt, B4-CON and B4-IoGAS1 consumed 16.8 ± 0.4 and 26.2 ± 1.9 g/L of the glucose at 72 h, respectively, and produced ethanol at 6.30 ± 0.41 and 9.98 ± 0.28 g/L at 72 h, respectively (Fig 6B). Thus, at pH 2.0 without the addition of salt, B4-IoGAS1 had a 56%-higher glucose consumption rate than B4-CON (0.364 ± 0.026 g/L/h vs. 0.234 ± 0.006 g/L/h, respectively), and a 58%-higher ethanol production rate (0.139 ± 0.004 g/L/h vs. 0.088 ± 0.006 g/L/h, respectively). The ethanol yields from consumed glucose after 72 h of fermentation at pH 2.0 without the addition of salt were similar in B4-CON (0.38 ± 0.02 g/g) and B4-IoGAS1 (0.40 ± 0.02 g/g). Moreover, under these conditions both strains produced no more than 1.18 g/L of glycerol and no more than 0.82 g/L of acetic acid from glucose at 72 h. These results indicated that even under low-pH conditions, glucose was efficiently converted to ethanol by the B4-IoGAS1 strain, that is, a strain in which *IoGAS1* was overexpressed.

Improvement of ethanol production by B4-IoGAS1 also was observed when conducting the fermentation in SCD-2 medium supplemented with 5% Na_2_SO_4_ at low pH (Fig 6C and 6D). At pH 2.5 with the addition of salt, B4-CON and B4-IoGAS1 consumed glucose at 24.8 ± 0.2 and 38.1 ± 0.5 g/L at 72 h, respectively, and produced ethanol at 7.63 ± 0.21 and 12.2 ± 0.3 g/L at 72 h, respectively (Fig 6C). Thus, at pH 2.5 with added salt, B4-IoGAS1 had a 54%-higher glucose consumption rate than B4-CON (0.530 ± 0.007 g/L/h vs. 0.344 ± 0.007 g/L/h, respectively), and 59%-higher ethanol production rate (0.169 ± 0.005 g/L/h vs. 0.106 ± 0.003 g/L/h, respectively). The ethanol yields from consumed glucose after 72 h of fermentation at pH 2.5 with added salt were similar in B4-CON (0.31 ± 0.00 g/g) and B4-IoGAS1 (0.32 ± 0.00 g/g), consistent with the higher level of glycerol (2.62 g/L) and lower level of acetic acid (0.48 g/L) generated by B4-IoGAS1. In contrast, a minimal amount of glycerol (less than 1.95 g/L) and no detectable acetic acid were excreted by B4-CON.

The superior fermentation performance of the *IoGAS1*-overexpressing strain was maintained when the external pH was reduced from 2.5 to 2.0 in the presence of 5% Na_2_SO_4_ (Fig 6D). At pH 2.0 with the addition of salt, B4-CON and B4-IoGAS1 consumed glucose at 13.7 ± 2.2 and 18.0 ± 0.9 g/L at 72 h, respectively, and produced ethanol at 3.27 ± 0.67 and 5.21 ± 0.09 g/L at 72 h, respectively (Fig 6D). A small amount of glycerol was produced by B4-CON (1.01 g/L) and B4-IoGAS1 (1.28 g/L), and no detectable acetic acid was produced by either strain. Thus, at pH 2.0 with added salt, B4-IoGAS1 had a 32%-higher glucose consumption rate than B4-CON (0.250 ± 0.013 vs. 0.190 ± 0.031 g/L/h, respectively) and a 60%-higher ethanol production rate (0.072 ± 0.001 vs. 0.045 ± 0.009 g/L/h, respectively). The ethanol yields from B4-CON and B4-IoGAS1 after 72 h fermentation in this severe fermentation medium were 0.24 ± 0.01 and 0.29 ± 0.01 g/g, respectively. Taken together, our findings indicated that under acidic and high-salt conditions, a strain with heterologous *IoGAS1* expression (B4-IoGAS1) demonstrated an improved ability to produce ethanol compared to the reference strain (B4-CON). Thus, the use of an *IoGAS1*-overexpressing strain may have several advantages for fermentation processes under acid and saline stress.

## Conclusions

*IoGAS1* was isolated from a genomic DNA library of *I*. *orientalis* and was identified as a novel gene required for a low-pH-tolerant phenotype in *S*. *cerevisiae*. The expression of *IoGAS1* in *S*. *cerevisiae* under the control of the *S*. *cerevisiae PGK* promoter significantly improved growth under acid stress (pH 2.0) and under combined acid-and salt-stress (pH 2.5, 7.5% Na_2_SO_4_) conditions. In addition, the *S*. *cerevisiae* strain expressing *IoGAS1* demonstrated a superior ability to ferment glucose to ethanol under the combined stress of low pH (between 2.0 and 2.5) and high salt concentration (5% Na_2_SO_4_). The results of this study strongly support the conclusion that the constitutive heterologous expression of the *IoGAS1* gene will prove useful for industrial applications to improve ethanol production as well as other bioproduction processes (e.g., lactic acid generation) under severe conditions of low pH and high salt concentration.

The amino acid sequence of the IoGas1 protein in *I*. *orientalis* was deduced and shown to have homology with other proteins. Similarity and homology were found with the Gas-family proteins of *S*. *cerevisiae* [43–45] and the Phr1 and Phr2 proteins of *C*. *albicans* [55,56]. These *Saccharomyces* and *Candida* proteins are members of the GPI-anchored GH72^+^ subfamily of β-1,3-glucanosyltransferases belonging to the GH72 glycosidase/transglycosidase family [72], suggesting similar functions of the IoGas1 protein in *I*. *orientalis*. Expression of the cloned *I*. *orientalis* gene in *GAS1*-deficient *S*. *cerevisiae* complemented the *Saccharomyces gas1Δ* mutant phenotype, showing that *IoGAS1* is both structurally and functionally similar to the *S*. *cerevisiae* gene. In particular, the *IoGAS1* gene was able to rescue *gas1Δ* sensitivity to CFW and SDS. Furthermore, we used site-directed mutagenesis to confirm that the IoGas1p Glu-161 and Glu-262 residues (which align with Glu residues essential for β-1,3-glucanosyltransferase activity in homologous proteins) were required for *IoGAS1* complementation of the morphological and stress-tolerance defects of the *Saccharomyces gas1* mutant. Northern blot analysis revealed that the endogenous expression of *IoGAS1*, like that of *CaPHR1* and *CaPHR2*, is modulated in response to ambient pH conditions. Although the precise cellular role of IoGas1p is still unknown, we postulate that this protein participates in the maintenance of cell wall integrity during environmental stress.
